# Thyroid Stimulating Hormone and Thyroid Hormones (Triiodothyronine and Thyroxine): An American Thyroid Association-Commissioned Review of Current Clinical and Laboratory Status

**DOI:** 10.1089/thy.2023.0169

**Published:** 2023-09-13

**Authors:** Katleen Van Uytfanghe, Joel Ehrenkranz, David Halsall, Kelly Hoff, Tze Ping Loh, Carole A. Spencer, Josef Köhrle

**Affiliations:** ^1^ Ref4U—Laboratory of Toxicology, Department of Bioanalysis, Faculty of Pharmaceutical Sciences, Ghent University, Ghent, Belgium.; ^2^ Department of Chemistry and Chemical Engineering, California Institute of Technology, Pasadena, California, USA.; ^3^ Cambridge University Hospitals Trust, Addenbrookes Hospital, Cambridge, United Kingdom.; ^4^ American Thyroid Association, Headquarters, Alexandria, Virginia, USA.; ^5^ Department of Laboratory Medicine, National University Hospital, Singapore, Singapore.; ^6^ Department of Medicine, University of Southern California, Los Angeles, California, USA.; ^7^ Charité–Universitätsmedizin Berlin, Corporate Member of Freie Universität Berlin and Humboldt-Universität zu Berlin, and Berlin Institute of Health, Institut für Experimentelle Endokrinologie, Berlin, Germany.

**Keywords:** standardization and harmonization, immunometric methods, mass spectrometry, thyroid hormones, thyrotropin, biological variation

## Abstract

**Background:**

Despite being the most performed laboratory endocrine investigation, the optimum use of thyroid tests (thyrotropin [TSH] and thyroid hormone [TH] measurement) is open to question and the interpretation of the results from these tests can be ambiguous. The American Thyroid Association (ATA) with its expertise support the endeavor of the U.S. Centers for Disease Control (CDC) and the International Federation of Clinical Chemistry and Laboratory Medicine (IFCC) to improve and maintain standardization and harmonization of thyroid testing. ATA mandated an international interdisciplinary working group panel to survey the status of thyroid testing by reviewing the recent literature to revise or update the criteria as needed in mutual agreement and to inform clinical care.

**Summary:**

This review represents the conclusions on the clinical use of current routine TSH and TH (thyroxine [T4] and triiodothyronine [T3]) assays, taking into account geographic differences in disease prevalence and clinical and laboratory practice among writing members. The interaction between physiological, pathophysiological, and pharmacological factors and thyroid assays can affect their measurements and confound result interpretation. These factors need to be considered in the clinical context of the patient for appropriate test ordering and result interpretation. Despite significant advances in laboratory methods over the past 50 years, routine thyroid assays remain susceptible to idiosyncratic analytical interference that may produce spurious results. Improved standardization needs to be demonstrated through ongoing international efforts before results from different assays can be considered equivalent. Emerging technology (e.g., mass spectrometry) shows promise for improved analytical performance, but more evidence of its clinical utility and improved throughput is required before it can be considered for routine use. Close clinical–laboratory collaboration is encouraged to overcome and avoid the pitfalls in thyroid testing as well as resolve clinically discrepant results. The evidence base supporting the conclusions of this review is summarized in four detailed online technical supplements.

**Conclusions:**

Over the past five decades, testing for TSH, T4, and T3 has evolved from manual radioisotopic immunoassays to nonisotopic multiplexed immunometric assays using highly automated equipment. Despite these technical advances, physicians and laboratorians performing these analyses must understand limitations of these methods to properly order tests and interpret results.

## PREFACE

This document aims to provide a state-of-the-art status report on the progress achieved in thyroid testing, including thyrotropin (TSH), thyroxine (T4), and triiodothyronine (T3). The target audience for this document includes general practitioners, endocrinologists, and laboratory professionals. It is not a systematic review or a guidance document addressing currently encountered diagnostic and clinical challenges related to these three key parameters of thyroid testing (Box 1). It does not cover the use of thyroid antibodies for investigating the etiology of thyroid dysfunction, or the use of thyroglobulin as a tumor marker for differentiated thyroid cancer or the investigation of primary congenital or acquired hypothyroidism.

**Box 1.** Key Clinical Points for Interpreting Thyroid Tests

• Nowadays, most thyroid tests are made on multianalyte automated immunoassay instrument systems.
• The same test reported by different manufacturer instruments can differ in absolute value and requires appropriate reference intervals for interpretation.
• Laboratories may change instruments and reference intervals without consulting or alerting physicians.
• Interfering factors in thyroid hormone tests are rare but can affect any thyroid test result. Interfering factors include heterophile antibodies (HAbs), analyte autoantibodies, high-dose dietary biotin, pharmaceuticals, and nutritional factors.
• HAb interference can affect multiple tests. When HAb interference with a test is detected, it should be noted in the patients' chart since it may also affect other laboratory investigations.
• Interference should be suspected when the test result is discordant with the clinical presentation of the patient. The laboratory may not have access to the clinical condition of the patient.
• The reference interval for a test typically represents the 95% confidence limits for a control population without thyroid disease.
• The between-person reference interval is typically wider than the within-person reference interval.
• Thyrotropin (TSH) represents a more sensitive reflection of thyroid status than free thyroxine (fT4), because of the log-linear TSH–fT4 relationship. However, TSH is only a valid measure of thyroid status if the hypothalamic-pituitary axis is intact.
• It may take weeks to months for the TSH measurement to accurately reflect thyroid status after an acute change.

Clinically relevant aspects of thyroid pathophysiology and the regulation of the hypothalamic-pituitary-thyroid-peripheral axis have recently been reviewed elsewhere.^1,2^ Generic abbreviations for thyroid hormones (THs), T4 and T3, are used in this text, if both total and free TH are meant. Total T4 (TT4) and total T3 (TT3) are used for total and free T4 (fT4) or free T3 (fT3) for free TH concentrations, respectively.

## HISTORY OF THYROID TESTING

Over the past 70 years, improvements in the sensitivity and specificity of thyroid tests have led to advances in detecting and treating thyroid disorders. Basal metabolic rate and bioassays were used to measure thyroid function for decades before the 1950s. Clinical laboratory-based TH measurement began in the 1950s with the measurement of the protein-bound iodine, a method that indirectly estimated the total (free+protein-bound) T4 (TT4) concentration,^3^ see Table 1.^4,5^

**Table 1.** Evolution of Thyroid Tests 1958–2021

Test	Development	Reference	Year
TT4 (estimate)	Measurement of protein-bound iodine	3	1958
Thyroxine binding globulin (estimate)	^131^I-T3 resin uptake	6	1963
TSH (1st generation)	RIA (sensitivity ∼1.0 mIU/L)	7	1965
fT4 (estimate)	^131^I-T4+equilibrium dialysis+MgSO_4_	8	1966
fT4 index	TT4+T3 resin uptake	9	1970
TRH stimulation	TRH-TSH (1st generation) testing	10	1972
TT4 and TT3	RIA methods	11–13	1970, 1971
fT4 and fT3 (estimates)	Analog and two-step immunoassays	14	1983
TSH (2nd generation)	Manual IRMA (functional sensitivity (0.1 mIU/L)	15	1986
TSH (3rd generation)	Manual ICMA (functional sensitivity (0.01 mIU/L)	16	1990
TT4	Isotope dilution/GC/TMS	17	1994
TT3	Isotope dilution/GC/TMS	18	1999
fT4 (direct)	Automated random access ICMA instruments	19–21	1988–2007
fT4 and fT3	Equilibrium dialysis/isotope dilution - liquid chromatography - tandem mass spectrometry (LC-TMS) reference method	21	2007
TSH (3rd generation)	Automated random access ICMA instruments	22	2017

See abbreviation list in Supplementary Data part for more details.

fT4, free T4; GC, gas chromatography; RIA, radio immunoassay; T3, triiodothyronine; T4, thyroxine; TMS, tandem mass spectrometry; TRH, thyrotropin releasing hormone; TSH, thyrotropin; TT3, total T3; TT4, total T4.

There was early recognition that abnormal TH binding proteins (primarily T4 binding globulin, TBG), which distribute TH to their target tissues including the brain, could distort the relationship between the total and the biologically active free TH, complicating the use of TT4 to assess thyroid function, for example, during pregnancy. As a result, indirect TBG assessments (uptake tests) were developed and used to adjust TT4 to provide an indirect estimate of free T4 (free T4 index (fT4I = TT4 + T3 resin uptake [T3RU] test).^9,23^ Current “uptake” tests, renamed TH binding ratios, called “T-uptakes,” mainly use automated immunological formulations and nonisotopic signals to assess available TBG binding sites relative to a “normal” reference that may be assigned a value of 1.00 or 40%, depending on the method.

Direct fT4 assays employing equilibrium dialysis or ultrafiltration to isolate the small (0.03%) biologically active fT4 fraction became available in the 1960s, but were technically complex and unsuitable to meet the increasing demand for thyroid testing.^8,24^ During the 1980s, the two-test fT4I (TT4 plus T3RU)^9^ began to be replaced by single test fT4 and fT3 immunoassays.^11–14,25^ The radioactive tracer (^125^I) was replaced by a nonisotopic signal, primarily chemiluminescence. Subsequently, free hormone (fT4 and fT3) immunoassay tests have become automated on multianalyte platforms and are currently used for most free TH testing.^19–21^ However, differences between the numeric values reported by different fT4 and fT3 methods negatively impact setting universal medical decision points and reference intervals for different patient populations.^17,18,21,26,27^

The first TSH assays, developed in the 1930s, were bioassay based and involved injecting pituitary extracts into guinea pigs and measuring histological changes in guinea pig thyroid glands.^28–31^ *In vitro* assays next appeared. These used cyclic adenosine monophosphate production by cultured thyroid follicular cells as a surrogate marker of the TSH concentration. Further evolution of TSH testing has reflected methodological improvements described for fT4 (Table 1). The first generation of TSH radio immunoassays developed in the 1960s lacked sufficient sensitivity to detect euthyroid or suppressed TSH and hence was only useful for diagnosing primary hypothyroidism.^7,32^

TSH assay “quality” has historically been defined by clinical sensitivity—the ability to discriminate between hyperthyroid and euthyroid TSH concentrations.^33^ This lack of sensitivity initially led to the use thyrotropin releasing hormone (TRH) administrations to obtain TSH concentrations into the measurable range for the detection of subclinical hypothyroidism and hyperthyroidism.^10,34^

During the 1980s, TRH testing was discontinued as TSH assay sensitivity was improved 100-fold by adopting immunometric assay (IMA) methodology and replacing the ^125^I tracer with a nonisotopic signal, primarily chemiluminescence.^15,16,35^ Since 2000, automated “3rd generation” IMAs with a functional sensitivity below 0.02 mIU/L have become the standard of care worldwide. With this limit of detection, the whole range of overt thyroid dysfunction from hyper- to hypothyroidism can be detected. However, a major limitation remains—the lack of assay specificity to distinguish between the bioactive TSH secreted in primary hypothyroidism versus the biologically inactive TSH isoforms typically secreted in central hypothyroidism.^36^

Besides the above-mentioned commonly used methods, two other techniques have been developed and implemented. These are immunochromatographic (lateral flow) point-of-care semiquantitative TSH assays,^37,38^ developed in the 1990s that can be used to screen for primary congenital hypothyroidism. The other technique is mass spectrometry-based methods. They were developed primarily for specific purposes such as primary reference in the diagnostic clinical field (e.g., as primary reference measurement procedures for standardization purposes and for resolution of discordant routine results in specialized centers). Note that new EU regulations regarding *in vitro* diagnostics mandate proper documentation if mass spectrometry (MS) methods are to be used for clinical diagnostics rather than research purposes.^39^

## THE CLINICAL UTILITY OF TSH MEASUREMENT

Serum TSH is one of the most frequently measured analytes in the outpatient setting. Thyroid disease is common and can in most cases be easily diagnosed based on using TSH measurement. In addition, the availability of high quality, sensitive and specific, and inexpensive TSH assays makes screening for thyroid disease cost-effective. Because of the log-linear relationship of TSH to fT4, deviations of TSH concentration from population-specific reference intervals represent the preferred initial test for evaluation of thyroid function.^40^

Understanding the indications for and limitations of TSH measurement and the interpretation of TSH results is essential for the practice of high-quality cost-effective medicine.^41^ TSH is used to screen newborns for primary congenital hypothyroidism and to screen adults at risk for thyroid disease. Note that primary congenital hypothyroidism is an endocrine emergency because delays in initiating treatment result in an irreversible loss of cognitive function.^42^ Accordingly, turn-around time from specimen collection to patient follow-up represents a major consideration in the implementation and quality assessment of newborn screening programs.

Interpreting the results for these apparently similar indications for TSH measurement is, however, very different (Box 2). Patient age presents an important factor that must be considered when interpreting TSH results. For example, TSH concentrations in newborns <3 days of age are difficult to interpret due the postnatal TSH surge. In newborns between the ages of 3 days and 1 month, serum TSH >20 mIU/L is generally considered as a clinical action limit requiring immediate action.^42^ At the other age extreme, in the elderly, the upper bound of the TSH reference interval progressively increases with age and should be reflected in laboratory reports and taken into consideration for clinical interpretation of TSH test results.^43–46^

**Box 2.** Points to Consider for Clinical Thyrotropin Measurements

Understanding TSH measurement and interpretation will lead to improvements in the quality and efficiency of endocrine care.
• Serum TSH measurement is the best test to screen for primary hypothyroidism in all age groups.
• TSH measurement alone is not sufficient for the diagnosis or treatment of patients with central hypothyroidism.^56^
• Interpretation of TSH values in patients with acute and/or intercurrent illness, for example, inpatients, is not straightforward and should take into account other patient factors.
• Age, sex, reproductive status, medications, ethnicity, iodine intake, and biological variation, but not circadian or circannual rhythms, are important variables to consider when interpreting TSH values.

As well as the initial clinical indication for which TSH is measured, other independent variables must be kept in mind when using and interpreting TSH measurements. Human thyroid function is affected by nutritional, environmental, geographical, genetic, and various (patho-)physiological endogenous or exogenous factors. These include population-specific iodine intake, patient age and sex, biological variation, reproductive status, ethnicity, and the assay method used (see Comparability and Quality Assessment of fT4 and TSH Assays section).^47–50^

Median TSH values tend to be higher in iodine-deficient populations than those found in iodine sufficient groups.^51–53^ This is reflected in geographically specific TSH reference intervals. Accordingly, population iodine intake should be considered when interpreting TSH values. During pregnancy, TSH concentrations are also affected. This results from the combined effects of high human chorionic gonadotropin (hCG) concentrations, especially during early pregnancy, and the fact that hCG is a weak thyroid stimulator because of shared homology with TSH. Hence, hCG will mimic TSH stimulation, T4 will be released, and TSH becomes suppressed to a variable extent. In addition to the aforementioned parameters, the reference interval for TSH during pregnancy varies also by trimester, fetal number, and geography (i.e., iodine intake and ethnicity) as well as the TSH assay used.^54,55^

TSH is lowest in the first trimester, lower in women with twins than in women with singleton pregnancies, is ∼0.4 mIU/L lower in African and Asian women, and is higher in women with insufficient iodine intake. Target TSH values for patients taking T4 are dependent on the clinical indication for hormone replacement; for instance, TSH suppression is often recommended for thyroid cancer patients post-thyroidectomy.

## THE CLINICAL UTILITY OF TH MEASUREMENTS COMPLEMENTING INTERPRETATION OF THE PRIMARY PARAMETER TSH

Serum TSH concentration is the single best biomarker to confirm a diagnosis and also the magnitude of primary thyroid disease as a consequence of the log-linear TSH–fT4 relationship.^1,57^ Although not necessary for the diagnosis of primary disease, T4 (and for specific constellations also T3) measurements are often added when TSH values fall outside the reference interval as this allows classification of thyroid disease into overt or subclinical^58,59^ and can direct therapeutic options accordingly (see “TSH and Thyroid Hormone Measurement: Clinical Algorithm” in Technical Supplementary Fig. S1).

TH measurement is also required to complement TSH measurement in a number of clinical situations (Table 2) such as pregnancy,^60^ intercurrent illness when TSH can be suppressed (the nonthyroidal illness syndrome^61,62^), and the initial treatment of hypo-^63^ or hyperthyroidism^64^ as the response of the pituitary-thyroid axis can be delayed after a change in thyroid status.^57^

**Table 2.** Common Physiological and Pharmacological Effects on the Biology of the Thyroid Hormone Axis

Cause	Drug	Effect
Inhibit TSH secretion	Dopamine L-dopa; glucocorticoids; somatostatin	↓ fT4; ↓ fT3; ↓ TT4; ↓ TT3; ↓ TSH
Inhibit TH synthesis or release	Iodine, lithium; 6-n-propyl-2-thiouracil, methimazole	↓ fT4; ↓ fT3; ↓ TT4; ↓ TT3; ↑ TSH
Inhibit conversion of T4 to T3	Amiodarone, glucocorticoids, propranolol, propylthiouracil; radiographic contrast agents	↓ TT3; ↓ fT3; ↑ rT3; ↓, ⇋, ↑ T4 and fT4; ⇋, ↑ TSH
Inhibit binding of T4/T3 to serum proteins	Salicylates, phenytoin, carbamazepine, furosemide, NSAIDs; heparin (*in vitro* effect)	↓ TT4; ↓ TT3; ⇋, ↑ fT4; ⇋, ↑ fT3; ⇋TSH
Stimulate metabolism of iodothyronines	Phenobarbital, phenytoin, carbamazepine, rifampicin	Thyroid axis should correct in euthyroid, requirement increase in hypothyroidism
Inhibit absorption of ingested T4	Aluminum hydroxide, ferrous sulfate, calcium salts, antacids, proton pump inhibitors, cholestyramine, colestipol; sucralfate, soybean preparations, kayexalate	↓ TT4; ↓ fT4; ↑ TSH (in hypothyroidism)
Increase in concentration of T4-binding proteins	Estrogen, clofibrate; opiates (heroin, methadone), 5-fluorouracil; perphenazine	↑TT4; ↑ TT3; ⇋fT4; ⇋TSH
Decrease in concentration of T4-binding proteins	Androgens, glucocorticoids	↓TT4; ↓ TT3; ⇋fT4; ⇋TSH

Adapted from Tietz Textbook of Laboratory Medicine Seventh Edition ISBN 9780323775724.^65^

TH, thyroid hormone.

While the term “thyroid function test” is commonly used, this term is only relevant to the untreated patient. This term is widely used to describe TSH and TH tests even though determinations of these parameters are often used to monitor hypothyroid patients on levo-T4 therapy with no innate thyroid function, patients with hypothalamic or pituitary disease (secondary hypothyroidism), or patients taking medications that affect the pituitary-thyroid axis. In addition, “thyroid function tests” are frequently used for diagnostic and therapeutic monitoring, for example, primary congenital hypothyroidism. Table 3 describes rare causes of perturbation of the hypothalamic-pituitary-thyroid axis.

**Table 3.** Rarer Causes of Perturbation of the Hypothalamic-Pituitary-Thyroid Axis

Axis perturbation	Prevalence	Mechanism	Reference
Secondary or tertiary (central) hypothyroidism	Central congenital hypothyroidism 1:13–16,000 Proportion of hypothyroid patients 1:1,000 Postpartum 1:20,000	Pituitary/hypothalamic	66–68
Autonomous TSH secretion	1:1,000,000	TSH secreting pituitary adenomas	69
TSH insensitivity syndromes	Not available	Failure of TSH to stimulate thyroid	70,71
TH resistance	1:19,000	Genetic variation in TH receptors	71,72

## T4, TOTAL, OR FREE TH ANALYSIS?

T4 is the prime TH that is measured. T4 is the main hormone exclusively produced and secreted by the thyroid gland (T4 to T3 ratio in thyroglobulin is ∼10:1).^73,74^ The majority of T3 (∼80%) is generated in extrathyroidal tissues by the two 5′-deiodination enzymes and thus subject to various (patho-) physiological influences, Circulating TH concentrations are affected by the concentration of serum TH binding proteins (TBG, transthyretin, albumin) as well as the hypothalamic-pituitary-thyroid axis.

For this reason, the free hormone hypothesis states that the unbound or free hormone fraction is likely to be a better marker for hormone action since this is the biologically active fraction. This is now widely accepted, at least for serum TH measurements, and serum fT4 should be measured in preference to TT4 despite its very low fraction of TT4 (0.03%).^75–78^

### T4 Metabolites

Although the measurement of T4 metabolites is technically feasible with modern mass spectrometric methods, clinical applications for the measurement of iodothyronine metabolites such as 3-monoiodothyronine (3-T1), 3,5-diiodothyronine (3,5-T2), and 3,3′-diiodothyronine (3,3′-T2), and for iodothyroacetic acids such as 3,3′,5-triiodothyroacetic acid (Triac, TA3) and 3,3′,5,5′-acid (Tetrac, TA4) have yet to be established. The potential clinical applications for TH metabolite profiling will require the development of “multiplex” methods wherein the concentration of multiple analytes can be ascertained simultaneously.^79–81^ The focus of this section will be on the measurement of T3 and reverse T3 (rT3).

#### T3 Measurement

T3 is predominantly protein bound (99.7%) and a direct measurement of fT3 is theoretically a better marker of thyroid function than TT3. However, the concentration of T3 in the circulation is lower than that of T4, and the binding affinity of T3 to carrier proteins in serum is weaker than that of T4. Consequently, fT3 measurements are more susceptible to interference by free fatty acids and drugs present in the circulation.^82^ As a result, the precision and reproducibility of fT3 immunoassays are less than that for fT4.

Consequently, many laboratories prefer to run TT3 assays rather than fT3 due to these concerns regarding fT3 immunoassay reliability. As T3 concentration can often be maintained within the reference interval in hypothyroidism, T3 measurement in patients with suspected hypothyroidism or with increased TSH is of limited clinical value.^83^ In hyperthyroidism, circulating T3 increases before T4. Consequently, the analysis of T3 can provide clinically relevant information in patients with suppressed TSH.^84^

The deiodinase enzymes 1 and 2 are responsible for the conversion of T4 to T3 and most of the T3 in circulation. The activity of these enzymes can be altered in patients with intercurrent illness, resulting in the low T3 concentrations that are characteristic of the nonthyroidal illness syndrome. Occasionally modification of the activity of deiodinase enzymes 1, 2, and 3 in patients on T4 replacement can also result in lower levels of circulating T3.^85^ In addition, circulating T3 can be reduced by increased activity of deiodinase enzyme 3 that can metabolize both T3 and T4.

As a T3 concentration below the reference interval is the hallmark of nonthyroidal illness, measurement of T3 is unlikely to be of diagnostic relevance in this context. However, T3 measurement can be useful in some clinical situations such as patients with low TSH and concomitant systemic or organ-specific disease. Here T3 measurement may help distinguish between hyperthyroidism and the nonthyroidal illness syndrome or to identify the coexistence of hyperthyroidism and intercurrent illness (Box 3).

**Box 3.** Clinical Utility of Triiodothyronine Measurement

• Total triiodothyronine (T3) measurement is preferred over free T3 measurement.
• Total T3 measurement may be useful in the evaluation of patients with suppressed TSH levels.
• Total T3 measurement is not helpful in the evaluation of patients with suspected hypothyroidism.
• Total T3 measurement can be helpful in:
○ the management of patients on T4 and T3 combination therapy for the treatment of hypothyroidism,
○ the monitoring of patients on suppressive doses of T4 for treatment of thyroid cancer^86^ and
○ the evaluation of patients on T4 treatment for hypothyroidism with suspected T4 to T3 conversion defects.
• Management of patients with Graves' disease, as alterations of T3/T4 ratio can be helpful in identifying patients whose disease may remit.^87^
• Reverse T3 (rT3) measurement is rarely useful, two very rare exceptions are the diagnosis of:
○ infrequent genetic thyroid syndromes and
○ consumptive hypothyroidism, a complication of unusual pediatric and adult tumors.

#### Reverse T3 Measurements

Unlike the active T4 metabolite T3, rT3 is an inactive metabolite of T4 as it does not bind or compete with T3 at the T3 receptor.^88^ Measurement of rT3 is widely cited in the lay press as a potential marker to guide T4 or T3 therapy, however, there is currently no evidence to support this application. Serum rT3 typically rises as T3 falls during nonthyroidal illness; consequently, measurement of rT3 adds little to this diagnosis unless nonthyroidal illness is confounding the diagnosis of central hypothyroidism.^89^

However, as rT3 assays are not widely available, measurement of T3 is more practical, cheaper, and as effective as measuring rT3 if nonthyroidal illness syndrome is suspected.^88,90^ Other current uses of rT3 analysis and the calculation of the serum rT3/T3 ratio are confined to the diagnosis of rare genetic thyroid conditions^91,92^ and the diagnosis of the rare consumptive hypothyroidism syndrome due to the overexpression of deiodinase enzyme 3.^93^ Except for these three uncommon situations, there is no need to measure rT3 in routine clinical practice.

## ANALYTICAL PERFORMANCE SPECIFICATIONS AND BIOLOGICAL VARIATION

Thyroid function tests, like most laboratory results, are susceptible to inaccurate measurement. Laboratory measurements can be affected by two different types of analytical error: (1) systematic error (also known as analytical bias) and (2) random error (also known as analytical imprecision). The so-called total error of measurement is a combination of these two parameters. Analytical errors can contribute to erroneous laboratory results, which, in turn, can lead to inappropriate disease classification and clinical decision making.^94^ To prevent inaccurate measurements affecting clinical decision making, analytical performance specifications (APSs) have been established to safeguard assay performance.^95^

APS can be used to optimize clinical utility in multiple aspects of laboratory testing, including the regulatory process for the approval of laboratory tests, proficiency testing of the individual laboratory, evaluation of laboratory methods, and monitoring the variability of lot-to-lot reagent changes.^96^ APS can be based on (1) clinical outcome studies and the impact on clinical decision- making (Models 1a and 1b, respectively); (2) biological variation (Model 2); (3) or state-of-the-art laboratory performance (Model 3).^95^ Of these, biological variation and state-of-the-art models are the mainstay of defining APS, as there are only limited studies that have examined the effects of APS on clinical outcomes or medical decisions.

Using biological variation data, the APS can be defined, as minimum, desirable, and optimum for both bias and imprecision^97,98^ (Table 4 and Supplementary Table S1). Simplistically, the APS model based on biological variation seeks to limit the analytical variability of a test (noise) relative to the biological variability (signal). Biological variation data are based on within-subject biological variation (CVi) that is the day-to-day fluctuation of a biomarker in an individual and the between-subject biological variation (CVg) that is the difference in physiological set-point among individuals within a population (Supplementary Fig. S2).

**Table 4.** Analytical Performance Specification Based on Biological Variation Data

Hormone	Within-subject biological variation (%)	Between-subject biological variation (%)		Minimum	Desirable	Optimal
TSH	18 (15–29)	36 (24–48)	Bias (%)	15	10	5
			Imprecision (%)	13	9	4
			Total error (%)	37	25	12
fT4	4.8 (4.8–9.5)	7.7 (7.5–12.1)	Bias (%)	3.5	2.3	1.2
			Imprecision (%)	3.7	2.5	1.2
			Total error (%)	9.5	6.3	3.2

Analytical performance specifications based on the meta-analysis of biological variation data (95% confidence interval of estimate in parentheses) curated by the European Federation of Clinical Chemistry and Laboratory Medicine Biological Variation Working Group (https://biologicalvariation.eu/, updated June 3, 2022).

The biological variation of TSH is much wider than that of fT4. Therefore, the desirable APSs for bias and imprecision of TSH are significantly larger than those for fT4 (Table 4).

Alternatively, the state-of-the-art APS can be derived from the evaluation and comparison of laboratory methods^99–104^ or using peer comparison data from proficiency testing programs.^105–107^ In general, the TSH assays in current clinical practice meet the desirable APS (based on biological variation) for imprecision. This may not be true for fT4 assays. Importantly, large intermethod biases are observed for both TSH and fT4 (see further in the text). This prevents the adoption of universal reference intervals and medical decision limits for thyroid tests.^101^ Moreover, the relationship between TSH and fT4 varies depending on the specific assay method used.^100^

### Biological variability of TSH and TH concentrations

The log-linear TSH–fT4 relationship illustrates both within- and between-subject biological variability in healthy individuals; this variability increases with both thyroid and nonthyroidal illnesses. Results from systematic studies in healthy mono- and dizygotic twins, repetitive sampling studies, and the testing of individuals or (sub-)populations in different regions have shown markedly narrower within-subject variability (e.g., during monthly sampling) than between-subject variations. Such studies reveal that heritability accounts for 30% to 65% of the variance in TSH, fT4, fT3, and the fT4 × TSH product. Population-based reference intervals for thyroid test variables are, therefore, much broader than the rather narrow within-subject variance and consequently are less useful for monitoring an individual patient.^108–113^

## COMPARABILITY AND QUALITY ASSESSMENT OF fT4 AND TSH ASSAYS

The results of hormone assays must be reliable given the high clinical impact on diagnosis, therapy, and monitoring of patients' health and disease. Thus, guarantee of their specificity, accuracy, and precision requires external reference points, standardized and certified reference materials, and regular documented proficiency testing. Compliance of the methods used in each laboratory with the International Guideline ISO 17511:2020^114^ on harmonization should be sought, as this will guarantee that the result for the patient samples is traceable to the SI units or the highest available standards.

### Comparability of results: The need for standardization/harmonization

**FIG. 1.**
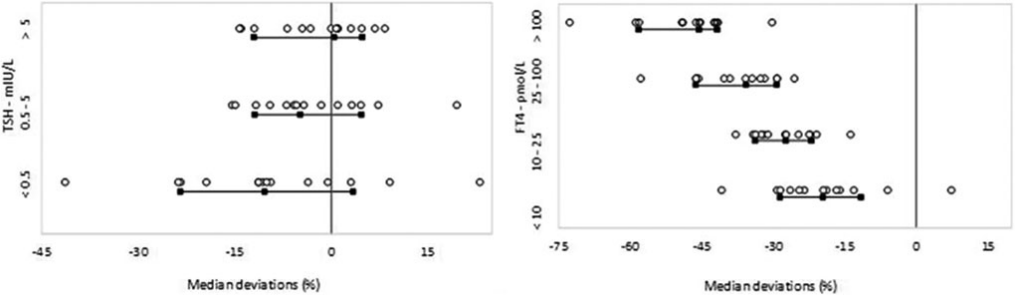
Status of comparability of results for both TSH and fT4 as documented in 2017 by the International Federation of Clinical Chemistry and Laboratory Medicine's Committee of Standardization of Thyroid Function Tests. Median deviations for each assay for a panel of ∼100 patient samples (serum) with concentrations spread over the entire measurement interval versus the reference are shown (the black lines and squares; the 15th, 50th, and 85th centiles, the vertical line; reference free thyroxine: candidate reference measurement procedure based on equilibrium dialysis—isotope dilution—mass spectrometry/reference thyrotropin: all procedure trimmed mean).^118^ For more details, we refer to the original publications.^22,115^ Modified and updated from Thienpont et al.^22^ and De Grande et al.^115^ with permission.

TH laboratory results ideally should be comparable over time, location, and independent of the method used. In 2017, the International Federation of Clinical Chemistry and Laboratory Medicine's Committee of Standardization of Thyroid Function Tests (IFCC C-STFT) documented the status of comparability of results for both fT4 and TSH assays from 13 of 15 assay manufacturers.^22,115–117^ Depending on the selected assay reagents and equipment, laboratory results may differ by up to 50% for both fT4 and TSH (Fig. 1). If method-specific reference intervals are not employed, the classification of a numerical test result may be different depending on the assay used.

In addition, the C-STFT also proposed a way to improve the current situation by standardizing fT4 and harmonizing TSH assays to reference measurement systems established according to ISO 17511.^114^ Their proof-of-concept study showed that for both fT4 and TSH, implementation of standardization/harmonization can reduce calibration differences between manufacturers.^22,115^ Note that the expected impact of standardization of fT4 values on patient results and reference intervals requires a carefully prepared transition as changes up to 30–50% are expected.^115^

### Quality assessment of analytical methods for TSH and TH

While the studies from the C-STFT date from 2017, information about current accuracy and quality of fT4 and TSH assays is very limited. Some information can be obtained from accuracy-based external quality assessment/proficiency testing programs.^119^ Additional information can be derived from outpatient data (e.g., the Noklus Percentiler^105,120^), or from anonymized electronic patient records. More research using different data sources is needed to monitor the accuracy of thyroid function tests.

A prerequisite for valid quality assessment is the commutability of the materials used. This means that the materials used for quality assessment should behave exactly as any patient sample in any given assay. Commercially available proficiency testing materials and quality control samples rarely replicate clinically relevant matrices and their variability. This has been demonstrated in a study for TSH reference materials, comparing TSH extracted from human pituitary tissue with recombinantly expressed (glycoengineered) TSH, both of which do not precisely match the sialylated TSH of hypothyroid patients.^121^

Similarly, fT4 reference material matrices are distinct from those of the serum or plasma of patients with nonthyroidal illness, disturbed lipid metabolism, or renal disease. Differences in matrix composition may require normalization procedures, which will limit within- or between-comparability of test results.

The continuing use of non-SI units (e.g., ng/dL) in clinical practice instead of correct SI terminology (mol/L) frequently creates confusion, especially if the concentration ranges of T4 are compared with those of T3. As TH circulates in the serum compartment of blood, results of TH measurement should be expressed as a (molar) amount present in a unit volume of serum.

## TECHNICALITIES OF THYROID TESTS

### Analytical challenges inherent to the glycoprotein nature of TSH

Two types of TSH assay have been used. These are bioassays and immunoassays, each of which has its own benefits and limitations. Bioassays measure the biological activity of TSH. Biological activity is affected by protein glycosylation, so changes in TSH glycosylation, which can occur, for example, in primary hypothyroidism, will be detected by bioassay. However, bioassays are time consuming, subject to many external variables, and expensive. Immunoassays in contrast are sensitive, specific, inexpensive, and widely available. Current immunoassays appear to be less affected by glycosylation changes in TSH, which occur in pituitary disease.^22,104^

They require appropriate assay calibrators and quality control samples. Typically, these are prepared by spiking of a reference material to a blank matrix. This has a major impact on the reference material used for spiking. Currently available TSH assays still use the international reference standards WHO IRP 80/558 or 81/565, both derived from cadaver pituitaries, which makes the post-translational modifications of this TSH different from those of TSH circulating in human serum. This may lead to different recognition by different mAbs used in various immunoassays and consequently differences in the results obtained. This is one of the reasons that harmonization of commercially available TSH methods is not yet achieved (see Comparability of result: The need for standardization/harmonization section).

For that reason, the C-STFT has developed a panel of certified single donor reference materials for TSH to be used for calibration and verification purposes. This panel includes a high variety of donors with different underlying diseases, hence covering a variety in TSH isoforms. Such calibrations can level off the impact of using just one distinctive isoform as in the WHO IRP material.

Mass spectrometry TSH assays are currently not clinically available as the development of these assays is technically demanding. Theoretically, mass spectrometric assays should be able to distinguish isoform differences due to alterations in glycosylation. However, from a practical point of view, this is limited by assay design and the current analytical sensitivity of mass spectrometers for the detection of low abundance glycoforms.

As well as these analytical issues, as previously discussed, the immunoreactivity of TSH may be affected by changes in glycosylation state such that the immunoreactive TSH concentration may not reflect bioactivity at the thyroidal TSH receptor.^122–124^

### Analytical challenges for the determination of free TH concentrations

The measurement of free TH represents a considerable analytical challenge as the vast majority of TH in the circulation is protein bound. Two methodologies are available for measuring serum-free THs: the first involves physical separation of free T4/T3 from its binding proteins using techniques such as dialysis or ultrafiltration,^78^ these are known as “direct” methods. The determination of the TH after physical separation is best performed using mass spectrometric methods. Direct methods are complex and unsuitable for high-throughput laboratory analysis and are usually only available in specialist referral laboratories.

While considered “gold standard” methods, it is important to note that the validity of these methods is only warranted when performed under conditions that minimally disturb the endogenous equilibrium between the free and bound hormone. Hence, rigorous control of key parameters is required. This is well described in the CLSI C45-A guideline.^125^ As with indirect methods, direct methods should be validated to minimize method-specific biases and to document assay-specific reference intervals.

The second class of methods (“indirect methods”) work on the assumption that the equilibrium between free and bound hormone will be maintained during analysis such that the immunoassay can be used to estimate the free fraction without physical separation of TH from its binding proteins.

Immunoassay methods for free TH have been widely implemented as they are amenable to high-throughput automation. Indirect methods have been shown to be reliable in most clinical situations, however, as comparative rather than absolute methods, they are not completely independent of changes in the concentration, the presence of competing ligands, or genetic variation in three major TH binding proteins. They are also subject to method-specific bias and require method-specific reference intervals.

The need to adopt method-specific reference intervals can be countered by using standardized fT4 tests (see further in the Comparability of result: The need for standardization/harmonization section). To achieve this goal, the IFCC C-STFT has developed a reference measurement system for fT4, including an fT4 reference measurement procedure, based on equilibrium dialysis—isotope dilution—liquid chromatography—mass spectrometry.^78,114^

Direct fT4 assays are recommended (1) when TH measurements by immunoassay are discordant with TSH or clinical findings, (2) in patients with known genetic binding protein abnormalities, and (3) other situations when immunoassay interference is suspected (Table 5).

**Table 5.** Causes of Artifactual Results in Thyroid Hormone Assays

Assay	Interference	Analyte	Effect	Laboratory mitigation	Estimate of prevalence	Reference
Immunoassay	Antireagent antibodies, Assay specific: Antianimal Ig Biotin/streptavidin Ruthenium heterophile antibodies	TSH	↓Blocking ↑Crosslinking	Method comparison—different antibody species/label. Immunosubtraction, e.g., PEG^a^ precipitation. Linearity/dilution studies. Withdraw biotin and resample. Heterophile blocking reagents.	Assay-dependent heterophile ∼0.4%	126–129,139,142
		fT4	Blocking↑	Method comparison—different antibody species/label. Equilibrium dialysis.	Unknown	
	Anti—hormone autoantibodies “macro TSH”^b^	TSH	↑	Immunosubtraction, e.g., PEG precipitation. Linearity/dilution studies. Gel filtration chromatography.	∼0.17%	130,138,142
	Anti—T4 antibodies	fT4	↑	Method comparison with two-step method/equilibrium dialysis. Immunosubtraction, e.g., PEG precipitation.	1.8%^c^	131
	Genetic variation in TSH p.(Arg75Gly) 25950606	TSH	↓	Assay-dependent method comparison.	Assay-specific allele frequency 0.13% (gnomAD v 2.1.1)	132
	T4 binding protein abnormalities Familial Dysalbuminemic Hyperthyroxinemia (FDH), Dystransthyretinemic hyperthyroxinemia (DTH)	fT4	fT4↑ TT4↑↑	Assay-dependent method comparison. Equilibrium dialysis. Alb, TRR gene sequencing.	Most common allele familial dysalbuminemic hyperthyroxinemia p.Arg242His 0.007% DTH p.Ala129Thr 0.003% (gnomAD v 2.1.1)^d^	133
Free T4 assay (direct and indirect assay)	Displacing agents					
	In vitro free fatty acid generation by heparin	fT4	fT4↑↑ TT4⇋	Predose sample, analyze immediately.	Not available	127,134,135
	Re-equilibration of T4 during assay	fT4	fT4↑↓	Predose sample, exchange medication.	Assay specific	134

^a^ Polyethylene glycol (PEG).^142^

^b^ Data from study of 1794 women of reproductive age @ heterozygotes have intermediate results.

^c^ Anti-T4 antibodies are frequently detected, however, relatively few cases are associated with assay interference that is typically only seen with one-step methods.

^d^ Autosomal dominant conditions.

Technical Supplements 2 and 4 elaborate in detail on the design of indirect routine methods and the comparison between indirect methods and direct methods using mass spectrometry for measurement of the free hormone fraction.

### Matrices for thyroid tests

In clinical practice, thyroid tests utilize samples such as whole blood, serum, or plasma that are collected by venipuncture—or in newborn screening—through heel prick or umbilical vein drainage. Dried blood samples obtained from capillary or whole blood require additional validation because of preanalytic variables, such as hematocrit, that can significantly affect results. Other biological fluids, for example, urine or saliva, obtained by noninvasive procedures or cerebral spinal fluid and tissue biopsies, which require intricate and invasive clinical procedures, are not used for the analysis of TH and TSH in current routine clinical practice.

Body fluid specimens utilized for thyroid test analyses may be used fresh or fresh-frozen and stored at −20°C, −40°C, −80°C, but repeated thaw–freeze cycles should be avoided. While T4 and T3, as small amphiphilic molecules are quite stable in specimens used in routine practice, the stability of the glycoprotein hormone TSH is limited and storage at room temperature and repeated freeze–thaw cycles should be avoided. The same applies for the determination of fT4 and fT3, although the molecules themselves are stable, TH binding proteins are affected by freeze–thaw cycles, which will affect the proportion of free hormone.

Most immunoassays used for thyroid test analyses are designed for a specific sample matrix, typically serum or plasma. The majority, but not all, assay kits currently provided by manufacturers will allow both types of specimens to be measured. Anticoagulants (e.g., EDTA, citric acid, and heparin) may interfere with immunoassay detection methods. Therefore, manufacturer's instructions for sample matrix required for immunoassays must be strictly followed. Both methods and instruments may be matrix sensitive.

### Confounders of thyroid tests

In most cases, a single measurement of TSH will accurately reflect the TH status. However, there are several situations when this is not the case. Clinicians should be aware of these shortcomings to avoid an incorrect diagnosis. The most common errors are due to misinterpretation of reference interval information due to selection of an inappropriate interval or lack of awareness of within-subject variation or assay imprecision. Pharmacological effects on the physiology of the thyroid axis are also relatively common with a wide variety of agents affecting thyroid test results as discussed above (Table 2). Some of these drugs may also directly interfere with components and principles used for the hormone assays.^136^

Rarely, more extreme analytical errors are present^137^ (Table 5). TSH immunoassays are prone to interference effects with endogenous antibodies directed against either TSH itself (“macro-TSH”^138^) or the assay reagents (heterophile or antianimal antibodies^139^) being the usual cause. Assay architecture-specific effects such as biotin interference have also been frequently reported.^126^

Unfortunately, as competitive immunoassay methods for fT4 are more complex than IMAs, they are more susceptible to both pharmacological (Table 2) and analytical errors (Table 4). This is usually due to the disruption of the delicate balance between free and bound T4 during assay due to aberrant binding proteins^140^ or the presence of T4 displacing agents^127^ such as free fatty acids generated by heparin administration.^135^ Autoantibodies directed against T4 are also a cause of assay interference in methods that coincubate the T4 tracer and anti-T4 antibody in the presence of serum components (“one-step” methods).^141^

While direct fT4 assays such as equilibrium dialysis methods are robust to most interferences that can affect immunoassay, they are still prone to displacement effects and hence rigorous attention is required when designing buffer components.

As matrix effects have the potential to distort the results of thyroid tests, they need to be minimized to guarantee sensitive, accurate, and precise hormone measurements irrespective of the method used. This includes matrix effects caused by the biological variability of samples undergoing TH analysis. MS-based methods typically compensate for these matrix effects and for sample loss during analysis by inclusion of an established amount of a stable isotopically labeled internal standards.

## CONCLUSIONS

Over the past five decades, testing for TSH and the TH (T4 and T3) has evolved from manual radioisotopic immunoassays performed in individual assay tubes to nonisotopic IMA tests made on highly automated immunoassay systems that provide substantial clinical utility. Analytes of interest can be quantified if appropriate reference measurement systems and certified standard materials are used. Within- and between-laboratory proficiency testing methods can provide insight into the performance of a particular method provided commutable samples are used. Unfortunately, these basic prerequisites are not yet implemented or regularly used.

Physicians and laboratorians must understand the limitations of TH measurement to properly order and interpret thyroid tests. There is a need for a stronger laboratory–clinician interface. In most geographic areas the laboratory receives a test request containing information relating to the patient identification that is missing clinical (Table 3) and pharmacological (Table 2) information and the circumstances prompting the test request. It can be critical for the laboratory to have this missing information given the various idiosyncratic analytical interferences that affect thyroid test reliability, discussed in this review. For their part, the laboratory should educate physicians regarding test limitations and interferences.

Furthermore, given the persistence of between-method differences, the laboratory should notify physicians before changing methods and reference ranges. The strengths and limitations of the major thyroid tests are discussed in this review and supplemental details are provided in the Technical Supplements.

Standardization, quality, performance, and harmonization of assays currently used in laboratory thyroid testing (TSH, T4, T3) need to be maintained and improved to enable exchange, application, and interpretation of test results within the medical community for rational and optimal evidence-based patient care. Development of emerging assay methodology (multiplexing, mass spectrometry, point-of-care tests, etc.) as well as computer- or artificial intelligence-aided evaluation and interpretation will require continuous communication and coordination to meet the demands of state-of-the-art patient care.

## Supplementary Material

Supplemental data
