# Supplementary material for: Thyroid Stimulating Hormone and Thyroid Hormones (Triiodothyronine and Thyroxine): An American Thyroid Association-Commissioned Review of Current Clinical and Laboratory Status
Source: Thyroid. 2023 Sep 13;33(9):1013–28. doi: 10.1089/thy.2023.0169 (PMC10517335; doi:10.1089/thy.2023.0169)
Supplement: Supplemental data [file Suppl_Data.docx]

Technical supplements

Technical Supplement 1

**Supplemental Table S1.**

**Mathematical definition of analytical performance specification based on biological variation:**

| **Analytical performance specification** | **Stringency** | **Formula** |
| --- | --- | --- |
| **Imprecision, (CVa, %)** | Minimum | 0.75 x CVi (%) |
|  | Desirable | 0.50 x CVi (%) |
|  | Optimum | 0.25 x CVi (%) |
| **Bias, (%)** | Minimum | 0.375 x (CVi^2^ + CVg^2^)^0.5^ |
|  | Desirable | 0.250 x (CVi^2^ + CVg^2^)^0.5^ |
|  | Optimum | 0.125 x (CVi^2^ + CVg^2^)^0.5^ |
| **Total error** |  | Bias + 1.65 x imprecision |

Where CVa is analytical variation, CVi is the within-subject biological variation, CVg is the between-subject biological variation.

**
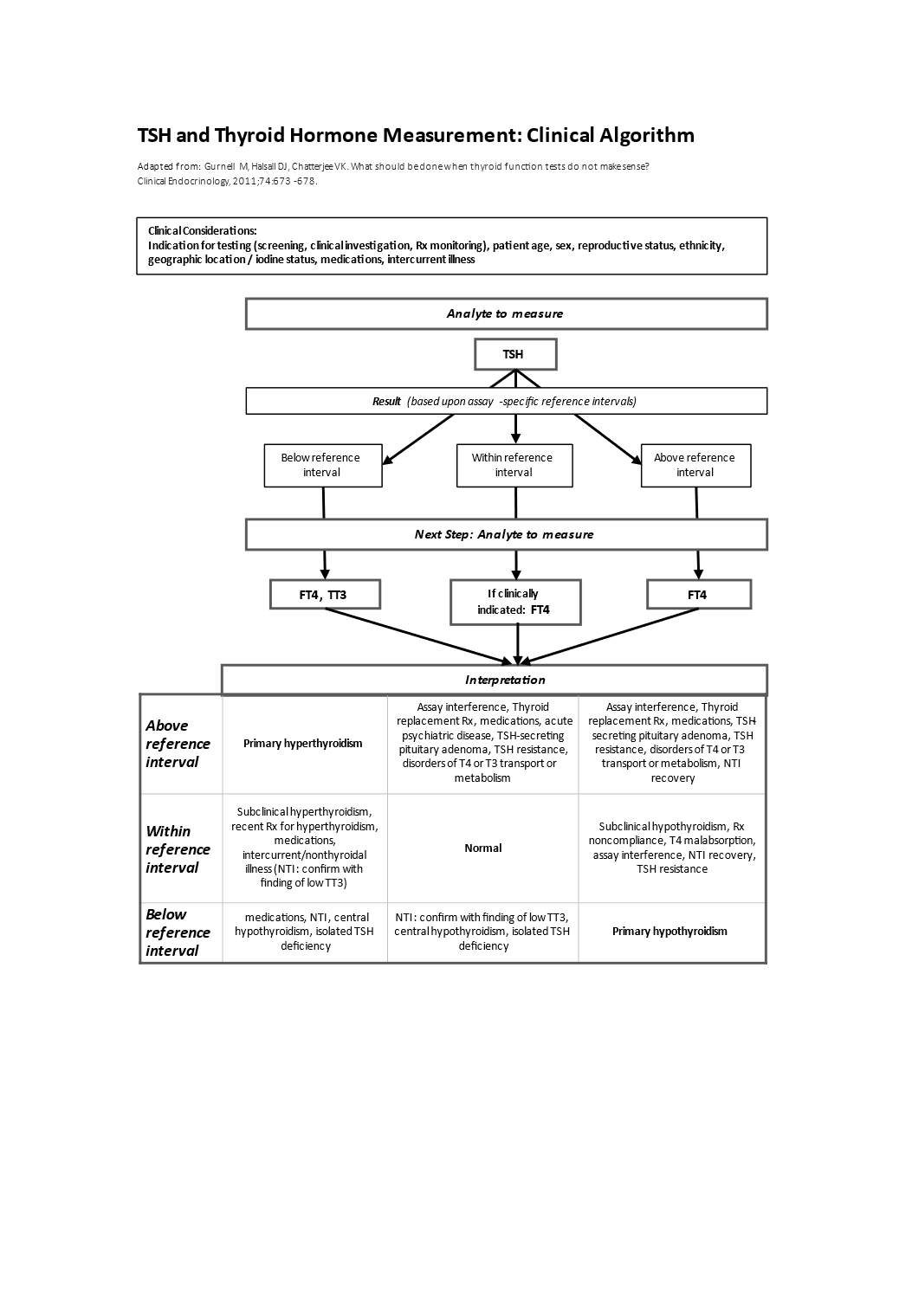
Supplemental Figure S1.**

**Supplemental Figure S2.**


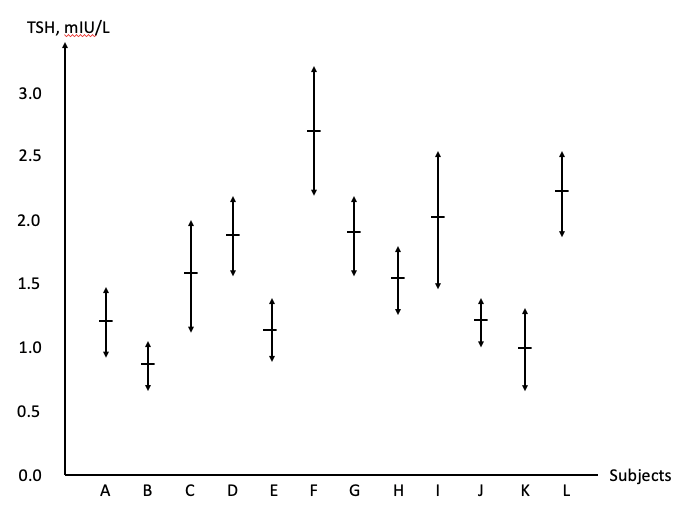
An illustrative example of biological variation for serum TSH in 12 hypothetical subjects (A-L). The vertical arrow represents the day-to-day biological variation within each subject (within-subject biological variation, CVi) whereas the horizontal bar represents the physiological set point for each subject. The between-subject biological variation (CVg) is the difference in the horizontal bar between the subjects.

**Technical supplement 2:**

**Antibody-based immunometric methods vs. mass spectrometry**

MS technology can unambiguously identify chemically related molecular fragments according to their mass/charge ratio and, at the same time, quantify them. In contrast, immunoassays exhibit inherent cross-reactivity with related epitope structures such as T4 and T3. T3 antibodies used must have minimal cross-reactivity with T4 to avoid overestimation of T3 measurement as T4 is at ~40-fold higher serum concentration. Several analytes of interest (> 20) can be quantified simultaneously in one run from a single low volume sample following suitable preanalytical workup ^I-IV^, while immunoassay methods typically require a sample aliquot for each component to assayed. MS methods require labor- and time-intensive preanalytical or complex online sample preparation to remove sample matrix components (e.g., phospholipids, specific ions) which can interfere with the highly sensitive MS detection of the analytes of interest. Direct immunometric assays are fraught with technical difficulties as the antigen-antibody interaction can be perturbed by components in the native test sample which are typically added directly to the assay mix. Immunoassays typically have a narrow dynamic range of ~ two orders of magnitude, while for MS assays the order of magnitude can span four to five orders thanks to the use of high-end mass spectrometers, or the flexibility of using variable (or higher) amounts of sample volume.

**Issues relevant for comparison and application of antibody-based immunometric methods vs. mass spectrometry in TFT**

A considerable advantage of immunoassay is that direct analysis of serum, plasma or DBS extracts is possible enabling speed, automation and high throughput of samples. No preanalytical sample workup or quality assessment of such procedures are needed for serum or plasma samples, but the matrix must be suitable for each assay kit design provided by the manufacturers. Serum, plasma, CSF, urine samples or DBS extracts (aqueous in case of TSH, organic solvents in case of T4 and T3) cannot be directly analyzed by every IA kit, if not validated for these matrices. T4 and T3 assay results are strongly affected by differences in sample matrix composition because of the high binding affinity of TH to their binding distributor binding proteins in blood (TBG, TTR, albumin), which deliver TH to their target tissues.

This may influence Ab binding kinetics during assay. Their presence, absence or variation in concentration (e.g., during pregnancy, in women using oral contraceptives, in patients with liver and/or kidney diseases, on hemodialysis, during acute phase reaction, under treatment by various drugs, etc.) markedly affects estimates of T4 and T3 concentration. This is relevant for total TH concentration but even more so for assays claiming to analyze free T4 or free T3 concentrations. Free TH concentrations amount to less than 1% of the total concentrations and thus are much more affected by any changes in the sample matrix (e.g., elevated non-esterified fatty acids, influence of anti-coagulants used, storage conditions, etc.) in addition to various clinical factors. ^V, VI^

The presence or absence of the 5’-iodine atom of iodothyronines conveys differences in solubility and charge of their 4’-hydroxyl residues at physiological pH, impacting on distinct (pre-)analytic properties of T4 and T3. This difference is relevant during sample preparation as pH changes of the specimen may affect both the immunoreactivity and cross-reactivity of Ab used in different assay kits.

A number of factors inherent to individual samples are known to interfere with immunoassay-based analysis of both TSH and T4 ^V^ such as biotin ^VII^, various frequently prescribed drugs, see tables in the main text ^VIII^ and a number of endogenous antibodies circulating in patients’ blood (anti-streptavidin, anti-ruthenium, anti-thyroid hormone autoantibodies, heterophile antibodies). In such cases, use of a different assay kit, method or vendor may solve the analytical problem and systematic sample dilution experiments may also help to identify the underlying interference. ^IX^

Such issues of interference and variation observed for direct sample application to the immunoassay reaction mixture are not directly relevant to the liquid chromatography – mass spectrometry (LC-MS) based TH analysis. MS technology identifies and quantifies chemically related molecule fragments in an unambiguous manner by sequential fragmentation and analysis of molecules of interest (e.g., T4, T3).

This series of charged molecular fragments generated and detected during the analytical MS process in multiple reaction monitoring (MRM) mode allows unequivocal identification and quantification of the desired precursor molecule. This method can be made even more selective if combined with prior chromatographic separation of chemically related analytes contained within the sample. However, isobaric compounds (ions with same mass number but different atomic number or composition) or isomers such as T3 and rT3 (same molecular formula, C15H12I3NO4, but different structures), if not separated via chromatography, will affect the quantification. Reliability can be increased by monitoring at least two fragments which allow for simultaneous detection of an identifier and quantifier fragment of the analyte of interest in MRM. Issues of cross- reactivities of Ab with related or chemically modified metabolites of analytes of interest or interference by drugs, metabolites or proteins originally contained in the sample will not affect LC-MS-based quantification.

Due to the increased sensitivity of LC-MS technology chemical derivatization of TH is no longer necessary before analysis. MS methods occasionally require labor- and time-intensive preanalytical sample preparation (see below) because application of complex mixtures to the MS analysis impairs detection sensitivity and precision.

Nevertheless, the disadvantage of pre-analytical sample preparation is outweighed by the possibility that a panel of several chemically related analytes of interest can be simultaneously quantified in a low volume sample using one single MS run. Recently, multiplex immunoassays for peptide-/protein-hormones have been developed. These have already been applied for analysis of cytokines with higher abundance in immunological and animal experimental settings. ^X^ These assays simultaneously use several specific antibodies, e.g., coupled to different beads, each detecting a specific analyte in the same sample mixture, thus saving sample volume and time. However, to date, multiplex assays are not yet available for TH T4 and T3 and other small molecule hormones such as steroids.

Differences in sample composition may affect analytical outcome. This will occur to a variable extent if samples or partially purified or enriched extracts are co-incubated with the test reagents in Ab-based immunometric assays or subjected to GC- or LC-MS. While the latter methods usually require application of sample extracts after pre-analytical workup, most immunoassays will tolerate direct use of small volumes of selected sample matrices such as serum or plasma. ^XI^

Ideally, for MS methods for each analyte to be determined (e.g., T4 and T3) a directly corresponding (synthetic) stable isotope labelled internal standard (SILIS) should be added before the pre-analytical sample workup for MS based identification and quantification. However, this provision is frequently neglected for time, cost and labor-saving reasons ^XII^. It is assumed, but not regularly proven, that these exogenously added standards (e.g., SILIS) precisely mimic the biochemical and analytical properties of the endogenous analyte of interest during the extraction and measurement procedure. This is a key prerequisite for isotope dilution methods to be used for the exact quantification of the analytes of interest. For example, an LC-MS method might be partially validated for the more abundant T4, while no such information is collected and available for T3, which occurs at much lower concentration and has different extraction properties to T4 during the preanalytical workup. Nevertheless, consistent application of SILIS renders it possible to compensate for sample extraction losses and analyte-specific differences in matrix effects on analyte ionization. ^I^

Consensus guidelines (i.e., CLSI 62-A) ^XIII^ are available for the validation of routine MS procedures. This includes, but is not limited to, a verification as to whether precision, bias and measurement range are fit for purpose (i.e., comply with pre-set analytical performance standards) and whether the analyte recovery is sufficient, the method is free from interference and is not influenced by sample matrix effects.

**Matrix-matched calibration and reference materials for immunoassays and LC-MS- based TH analytics**

Preparation of matrix-matched calibration samples can minimize but not exclude matrix effects both in LC-MS- and immunoassay-based analytical methods. However, lipemic, hemolytic, uremic, icteric samples and those with altered protein content cannot be comprehensively mimicked. The use of artificial protein matrices or synthetic surrogate matrices with, for example, known protein, lipid and electrolyte content, may improve analytical precision ^XIV^, but this is yet to be routinely implemented in clinical laboratories. For immunoassays, the commutability of any matrix-based calibrator (whether the matrix-based calibrator behaves like a real patient sample) may jeopardize the accuracy of the method. Consequently, careful validation is required.

**Technical Supplement 3**

**Dried Blood Spots (DBS) for newborn screening (NBS)**

TSH measured from dried whole blood samples (DBS), obtained by heel pricks of neonates on day 3-5 after birth are the most commonly used sample matrix and analyte used to screen for primary congenital hypothyroidism, although some newborn screening programs also advocate TT4 and TBG measurement to also detect central congenital hypothyroidism (see technical supplement 2 for more details and advice).

Immunochromatographic (lateral flow) point-of-care semi-quantitative TSH assays can be used to screen for hypothyroidism. Although the limit of detection of point-of-care TSH measurement methods is not as low as that of commonly used laboratory TSH assays, these inexpensive devices are able to quickly and conveniently detect the increased serum TSH found in patients with untreated and incompletely treated hypothyroidism. ^XV^

**Factors affecting and interfering with dried blood spot (DBS)-based new born screening (NBS) for** **primary congenital hypothyroidism and alternatives to the DBS-based NBS approach**

Newborn hematocrit is the most critical factor influencing the analytical result for TSH. Hematocrit, typically in the range between 55% and 68% in newborns, not only affects diffusion characteristics of the blood drops on the filter paper but also shows marked variations (from 30 to 70%) dependent on ethnic, geographic, environmental and concomitant pathophysiological factors. ^XVI^ If using TSH concentration as the diagnostic parameter for NBS, a high hematocrit may lead to false negative results, while low hematocrit in regions of pandemic anemia may return false positive TSH values. Hematocrit has two major effects. Firstly, samples with high hematocrit have a higher viscosity, so they will spread less. As the hematocrit increases, the same area of a blood spot will contain an increased volume of blood. Secondly, and in opposition, samples with high hematocrit, will have proportionally less serum in the same volume of blood. This is relevant for analytes such as TSH which are solely present in the serum compartment of blood. Because, for the same volume of sample, there will be less serum, hence lower absolute amounts of TSH. These two effects may counteract each other in an analyte dependent manner.

For TSH, Butler demonstrated that the second effect is dominant. Recent secular and medical trends in obstetrics and perinatal medicine (“timed delivery”, increased rates of caesarian section, ambulatory parturition, etc.) may require adaptation of CH NBS procedures to these changes. Established cut-off values for TSH or T4 reference intervals, based on postnatal day 3-5, when the parturition induced transient TSH and T4 surges have passed, will not be applicable for heel prick DBS samples taken from newborns during the first two postnatal days. The numbers of false negative and false positive tests will increase and require recalls for regular venipuncture and combined TSH and T4 confirmatory testing during the first two weeks. This may result in increased cost of CH NBS programs and potentially decrease their precision and clinical effectiveness. Thus, an alternative NBS approach using larger volume cord blood samples collected at birth and direct hormone analysis in serum or plasma, avoiding DBS preparation, has been proposed and implemented in several successful NBS programs, e.g., in India. ^XVII^ Birth- related TSH increases in newborn serum start around ½ hour after birth. Nevertheless, CH NBS using cord blood sample will require higher TSH cut-offs (e.g., TSH > 40 mIU/L). However, no correction factors are needed to extrapolate from DBS analysis to blood concentrations. Preanalytical issues of DBS preparation and extraction are no longer relevant as are issues of instability TSH (or T4) on DBS cards. Point-of-care immunochromatographic TSH assays represent an alternative method for NBS. The advantages of rapid, on-site TSH screening are that results are available at the bedside in minutes and tests can be performed without the need for tightly controlled specimen preparation protocols (https://[www.thyroid.org/wp-content/uploads/publications/lab-](http://www.thyroid.org/wp-content/uploads/publications/lab-)services/ata-poc-thyroid-management.pdf).

Elevated humidity, light exposure and temperature variations may result in T4 degradation and TSH

instability in DBS specimen. ^XVIII,XIX^ Seasonal variations have been observed for both TSH and T4 values in primary congenital hypothyroidism NBS programs, with TSH and T4 concentrations higher during the cold compared to the warm season. Depending on the primary parameter used, more false positive cases are observed for TSH during winter and a higher rate of false positive cases for T4 during summer. ^XVIII^

Technical Supplement 4:

**The design of routine free thyroid hormone laboratory methods.**

Three types of immunoassay for free hormones measurement are currently available, which use either a labeled hormone analog or antibody. These are the one-step, two-step labeled hormone and the back titration methods. These are shown graphically in the figure below, extracted from. ^XX^


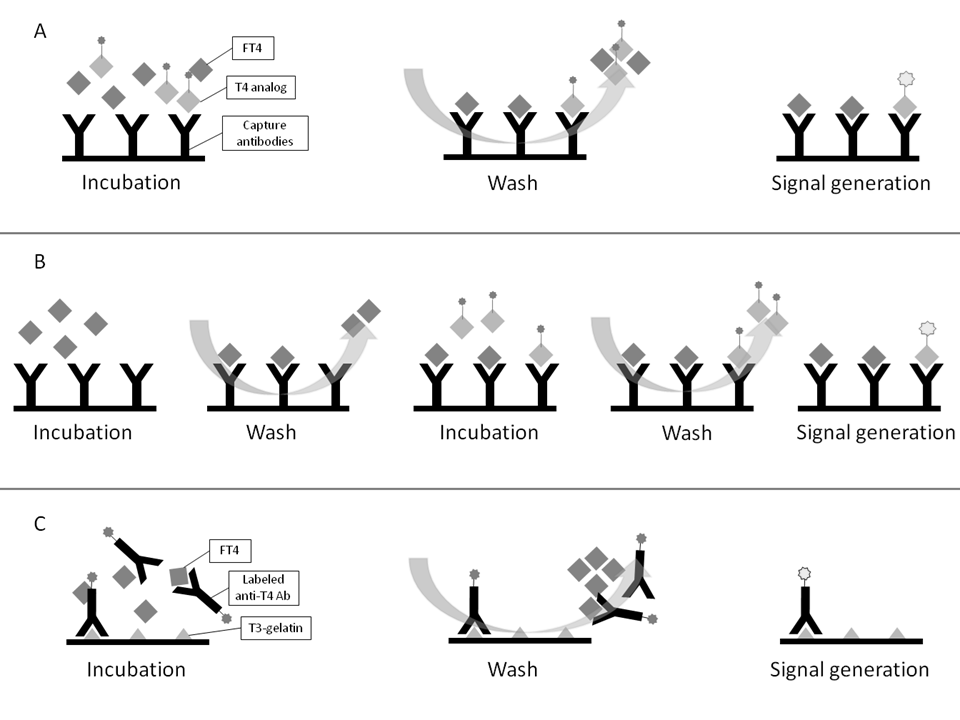


(A) The one-step analog design; the patient sample is simultaneously incubated with a labeled T4

analog which competes for binding to an immobilized anti-T4 antibody, followed by a wash step before the signal is recorded. (B) The two-step analog design; an additional wash step is performed before the addition of a labeled T4 analog. (C) The labeled-antibody design; T3-gelatin is immobilized and competes with the free T4 in the sample for binding to a labeled anti-T4 antibody in the reagent. In all these designs, the signal generated is inversely proportional to the free TH concentration.

Technical Supplement 5

**Requirements for assessment of the analytical validity of TH and TSH immunoassays**

The best validation of the performance of both an immunoassay or a routine LC-MS method is by comparison with a reference measurement procedure, as referred to in the section comparability and quality assessment of FT4 and TSH assays. Before this can be done, the basic analytical validity in terms of assay design, linearity, limit of detection, precision, cross-reactivity, etc. should be assessed. The Clinical and Laboratory Standards Institute (CLSI) has published multiple internationally accepted guidelines and protocols on how to do so, of relevance

are the CLSI C45-A guideline for free hormone assays and the CLSI C62Ed2 for liquid-chromatography mass spectrometry methods. ^XXI,XXII^

Unknown cross-reacting compounds in samples may not be identified by immunoassay and return false values, whilst adequately established LC-MS methods will not be affected cross-reacting species. A prerequisite for competitive immunoassay is a labelled analogue of the desired analyte which has similar immunological properties to the analyte in question. Chemical modification of analytes such as tagging by chromophores and iodination are often used to produce assay tracers. These modifications may affect the tracers’ immunoreactivity and cross reactivity. For T4 and T3 this is not an issue if the naturally occurring 127-I substituents are replaced by the radioactive isotope 125-I. However, any modification of the 4’-OH group or alanine side chain function of iodothyronines, which is typically employed in non-radioactive TH IA, may affect Ab recognition and reactivity.

**References to the technical supplements**

I. Köhrle J, Richards KH. Mass Spectrometry-Based Determination of Thyroid Hormones and Their Metabolites in Endocrine Diagnostics and Biomedical Research - Implications for Human Serum Diagnostics. Exp Clin Endocrinol Diabetes. 2020;128(6-07):358-374, doi:10.1055/a-1175-4610

II. Richards KH, Monk R, Renko K, et al. A combined LC-MS/MS and LC-MS(3) multi-method for the quantification of iodothyronines in human blood serum. Anal Bioanal Chem. 2019; 411:5605-5616; doi: 10.1007/s00216-019-01941-9.

III. Jongejan RMS, Klein T, Meima ME, et al. Mass Spectrometry-Based Panel of Nine Thyroid Hormone Metabolites in Human Serum. Clin Chem. 2020; 66:556-566; doi: 10.1093/clinchem/hvaa022

IV. Martínez Brito D, Leogrande P, de la Torre X, Botrè F. Optimization of a method to detect levothyroxine and related compounds in serum and urine by liquid chromatography coupled to triple quadrupole massspectrometry. J Pharmacol Toxicol Methods. 2022;115:107169. doi: 10.1016/j.vascn.2022.107169.

V. (CLSI) CaLSI 2022 CLSI document C62-A: Liquid Chromatography-Mass Spectrometry Methods. 2nd Edition. CLSI, Wayne, PA.

VI. Favresse J, Burlacu MC, Maiter D, et al. Interferences With Thyroid Function Immunoassays: Clinical Implications and Detection Algorithm. Endocrine Reviews. 2018;39(5):830-850, doi:10.1210/er.2018-00119

VII. Razvi S, Bhana S, Mrabeti S. Challenges in Interpreting Thyroid Stimulating Hormone Results in the Diagnosis of Thyroid Dysfunction. J Thyroid Res. 2019;2019(4106816, doi:10.1155/2019/4106816

VIII. Ylli D, Soldin SJ, Stolze B, et al. Biotin Interference in Assays for Thyroid Hormones, Thyrotropin and Thyroglobulin. Thyroid. 2021;31(8):1160-1170, doi:10.1089/thy.2020.0866

IX. Burch HB. Drug Effects on the Thyroid. N Engl J Med. 2019;381(8):749-761, doi:10.1056/NEJMra1901214

X. Kristensen GB, Rustad P, Berg JP, et al. Analytical Bias Exceeding Desirable Quality Goal in 4 out of 5 Common Immunoassays: Results of a Native Single Serum Sample External Quality Assessment Program for Cobalamin, Folate, Ferritin, Thyroid-Stimulating Hormone, and Free T4 Analyses. Clin Chem. 2016;62:1255-1263; doi: 10.1373/clinchem.2016.258962

XI. Mountjoy KG. ELISA versus LUMINEX assay for measuring mouse metabolic hormones and cytokines: sharing the lessons I have learned. J Immunoassay Immunochem. 2021;42(2):154-173, doi:10.1080/15321819.2020.1838924

XII. Caruso B, Bovo C, Guidi GC. Causes of Preanalytical Interferences on Laboratory Immunoassays – A Critical Review. EJIFCC 2020;31(1):70-84

XIII. Rappold BA. Review of the Use of Liquid Chromatography-Tandem Mass Spectrometry in Clinical Laboratories: Part I-Development. Ann Lab Med 2022;42(2):121-140, doi:10.3343/alm.2022.42.2.121

XIV. Ko AY, Yang JY, Kim D, et al. Quantification of triiodothyronine and thyroxine in rat serum using liquid chromatography tandem mass spectrometry. J Pharm Biomed Anal 2021;195:113840; doi: 10.1016/j.jpba.2020.113840

XV. American Thyroid Association. Point-of-Care Thyroid Diagnostics and Thyroid Disease Management. https://www.thyroid.org/wp-content/uploads/publications/lab-services/ata- poc-thyroid-management.pdf. (Last accessed on October 16th 2022, 2022).

XVI. Butler AM, Charoensiriwatana W, Krasao P, et al. Newborn Thyroid Screening: Influence of Pre- Analytic Variables on Dried Blood Spot Thyrotropin Measurement. Thyroid 2017;27(9):1128-1134, doi:10.1089/thy.2016.0452

XVII. Desai MP, Sharma R, Riaz I, et al. Newborn Screening Guidelines for Congenital Hypothyroidism in India: Recommendations of the Indian Society for Pediatric and Adolescent Endocrinology (ISPA–) - Part I: Screening and Confirmation of Diagnosis. Indian J Pediatr 2018;85(6):440-447, doi:10.1007/s12098-017-2575-y

XVIII. McMahon R, DeMartino L, Sowizral M, et al. The Impact of Seasonal Changes on Thyroxine and Thyroid-Stimulating Hormone in Newborns. Int J Neonatal Screen 2021;7(1), doi:10.3390/ijns7010008

XIX. Lando VS, Batista MC, Nakamura IT, et al. Effects of long-term storage of filter paper blood samples on neonatal thyroid stimulating hormone, thyroxin and 17-alpha-hydroxyprogesterone measurements. J Med Screen 2008;15(3):109-11, doi:10.1258/jms.2008.007086

XX. Loh TP, Leong SM, Loke KY, et al. Spuriously elevated free thyroxine associated with autoantibodies, a result of laboratory methodology: case report and literature review. Endocr Pract 2014;20(8):e134-9, doi:10.4158/EP14059.CR

XXI. Thienpont LB, Bunk DM, Christofides ND et al. Measurement of free thyroid hormones. Clinical and Laboratory Standards Institute Wayne, PA 19087 USA; 2004.

XXII. Clarke W, Molinaro RJ, Bachmann LM, et al. CLSI document C62-A: Liquid Chromatography-Mass Spectrometry Methods. Clinical and Laboratory Standards Institute: Wayne, PA 19087 USA; 2014.

**Abbreviation list:** Van Uytfanghe et al. Thyroid Tests

| **Abbreviation** | **Explanation** |
| --- | --- |
| **Ab** | Antibody |
| **APS** | Analytical Performance Specifications |
| **CSF** | Cerebro-Spinal Fluid (liquor) |
| **CVi** | within-subject Biological Variation |
| **CVg** | between-subject Biological Variation |
| **DBS** | Dried Blood Sample |
| **DTH** | Dystransthyretinemic hyperthyroxinemia |
| **FS** | Functional Sensitivity; concentration that results in a CV=20% (or some other predetermined % CV) |
| **EQA/PT** | External quality assessment/proficiency testing |
| **FDA** | Familial dysalbuminemic hyperthyroxinemia |
| **FT3I** | Free T3 Index |
| **FT3** | Free T3 |
| **FT4** | Free T4 |
| **FT4I** | Free T4 Index |
| **GC** | Gas Chromatography |
| **1st G** | 1st Generation TSH FS (~1-2 mIU/L) |
| **2nd G** | 2nd Generation TSH FS (~0.1-0.2 mIU/L) |
| **3rd G** | 3rd Generation TSH FS (~0.01-0.02 mIU/L) |
| **hCG** | human Chorionic Gonadotropin |
| **IA** | Immunoassay |
| **ICMA** | Immunochemiluminometric assay |
| **IMA** | Immunometric assay |
| **IRMA** | Immunoradiometric assay |
| **LFT; LFD** | Lateral Flow Test, or lateral flow device (LFD); rapid, antibody-based immunochromatographic assay used for qualitative bed-side point of care resting |
| **LC** | Liquid Chromatography |
| **LC-MS** | Liquid Chromatography - Mass Spectrometry |
| **MRM** | Multiple Reaction Monitoring |
| **MS** | Mass Spectrometry |
| **NBS** | Newborn screening |
| **NEFA** | Non-Esterified Fatty Acids |
| **PBI** | Protein Bound Iodine |
| **PEG** | Polyethylene glycol |
| **RIA** | Radioimmunoassay |
| **rT3** | reverse-Triiodothyronine, 3,3',5'-Triiodo-L-Thyronine |
| **T3RU** | T3 Resin Uptake |
| **TBG** | Thyroxine Binding Globulin |
| **TFT** | Thyroid function tests |
| **TT** | Thyroid tests; blood test which use any component of the hypothalamus-pituitary-thyroid-periphery |
| **TH** | Thyroid Hormone |
| **THBR** | Thyroid hormone binding ratio: thyroid function test that measures the "uptake" of T3 or T4 tracer by thyroid-binding globulin (TBG) in a given serum sample. |
| **TMS** | Tandem Mass Spectrometry |
| **TRH** | Thyrotropin Releasing Hormone |
| **TTR** | Transthyretin |
| **TSH** | Thyroid Stimulating Hormone / Thyrotropin |
| **T4** | Thyroxine, 3,3',5,5'-Tetraiodo-L-Thyronine |
| **T3** | Triiodothyronine, 3,3',5-Triiodo-L-Thyronine |
| **T3RU** | T3 resin uptake |
| **TT3** | Total Triiodothyronine |
| **TT4** | Total Thyroxine |
| **Guideline**  **Abbreviations** |  |
| **ISO 17511:2020** | International Guideline on harmonization of in vitro diagnostic medical devices |
| **EQA/PT** | External Quality Assessment (EQA) / Proficiency Testing (PT) process by the College of American Pathologists (CAP) |
| **WHO IRP 80/558** | International Reference Preparation of TSH prepared from pituitaries of human cadavers |
| **CLSI C45-A** | Clinical and Laboratory Standards Institute (CLSI) guideline for measurement of free thyroid hormones |
| **IFCC C-STFT** | International Federation of Clinical Chemistry and Laboratory Medicine’s Committee of Standardization of Thyroid Function Tests |

**Conflict of Interest Disclosures:**

| First Name | Last Name | Financial Disclosure Form? | FDF Date Reviewed  /Confirmed | Disclosures Noted | Is this relationship relevant? |
| --- | --- | --- | --- | --- | --- |
| Josef | Köhrle | Yes | 3/12/2023 | None | n/a |
| Joel | Ehrenkranz | Yes | 3/11/2023 | None | n/a |
| David | Halsall | Yes | 3/7/2023 | None | n/a |
| Kelly | Hoff | Yes | 3/1/2023 | None | n/a |
| Tze Ping | Loh | Yes | 2/28/2023 | None | n/a |
| Carole | Spencer | Yes | 1/19/2023 | None | n/a |
| Katleen | Van Uytfanghe | Yes | 3/10/2023 | None | n/a |
